# VISION: View-specific integrated segmentation-classification framework for accurate brain tumor detection in MRI scans

**DOI:** 10.1371/journal.pone.0332395

**Published:** 2025-10-15

**Authors:** Namya Musthafa, Mohammad Mehedy Masud, Qurban Memon, Moomal Farhad

**Affiliations:** 1 College of Engineering, United Arab Emirates University, Al Ain, United Arab Emirates; 2 College of Information Technology, United Arab Emirates University, Al Ain, United Arab Emirates; Università degli Studi di Napoli Federico II: Universita degli Studi di Napoli Federico II, ITALY

## Abstract

Brain tumors are an increasing global health concern, and accurate diagnosis is essential for improving patient outcomes. Although existing Magnetic Resonance Imaging (MRI)-based machine learning utilizes computer vision for tumor diagnosis, these methods are limited. They either focus solely on segmentation, which does not facilitate tumor detection, or on classification, which fails to identify tumor boundaries. To overcome these limitations, we propose the “View-specific Integrated Segmentation-Classification” (VISION) framework, designed for more accurate brain tumor diagnosis by integrating both segmentation and classification processes. The VISION framework introduces two novel components: (1) a View Classifier that determines MRI orientation (axial, coronal, or sagittal), and (2) a view-specific integrated network combining a customized segmentation model with a classification header. This architecture simultaneously identifies tumor boundaries (segmentation) and detects tumor presence (classification). We evaluated our approach using publicly available data and compared it against state-of-the-art MRI-based tumor diagnosis techniques. The VISION framework outperformed existing methods, achieving a Dice score of 0.89, an IoU of 0.87, and an F1 score of 0.98 while maintaining competitive computational efficiency. The proposed VISION framework offers a robust solution for brain tumor diagnosis by integrating view classification, segmentation, and detection into a unified system. Its high accuracy and efficiency demonstrate significant potential for clinical applications in improving tumor diagnosis and treatment planning.

## Introduction

The incidence of positive tumor cases has increased significantly in recent decades. In 1999, the American Cancer Society reported [1] 16,800 diagnosed cases of brain tumors, resulting in nearly 13,000 deaths. This underscores the urgent need for improved detection and treatment methods. According to a recent report on cancer statistics [2], in 2022, the United States saw 1,958,310 new cancer cases and 609,820 cancer deaths. Additionally, global cancer statistics [3] for 2022 indicate that there were almost 20 million new cancer cases and around 10 million cancer deaths worldwide. The study also predicts that by 2050, the number of new cancer cases could reach 35 million annually, representing a 77% increase compared to 2022. Accurate diagnosis is crucial because incomplete or incorrect diagnoses can lead to inappropriate treatment [4].

Generally, tumors can be classified based on their nature, origin, grading, and progression stages [5]. Tumors within the brain or spinal cord can interfere with normal brain function, and these effects depend on their classification. Hence, detecting tumors accurately and delivering precise treatment is critical to improving patient outcomes, which is why progress in imaging and diagnostic techniques is extremely important. The benign and malignant tumors are classified according to their growth rate and aggressiveness. Tumors originating directly in the brain are called primary tumors, whereas secondary tumors develop elsewhere before spreading to the brain. Types of tumors are also based on their origin; for example, pituitary tumors are found in the pituitary gland, glioma arise from glial cells, whereas meningioma arises from the meninges.

Manual diagnosis of brain tumors by human experts poses some challenges. First, it is influenced by several cognitive biases, as reported in the studies [4,6,7], which are discussed below. Anchoring bias involves fixating on an initial impression, while framing bias is swayed by how clinical information is presented. Availability bias leads to a tendency to favor common diagnoses, and confirmation bias drives radiologists to seek evidence that supports their initial hypothesis. Satisfaction of search and premature closure involves stopping further investigation once a diagnosis is made, potentially leading to the missed detection of additional abnormalities. Outcome bias reflects a tendency to favor diagnoses suggesting a better prognosis, and zebra retreat describes the hesitation to report rare conditions despite supporting evidence. These biases can impact diagnostic accuracy and patient care. Second, manual diagnosis is labor-intensive and time-consuming, requiring careful examination of each MRI slice. This task becomes particularly daunting when dealing with large volumes of data and the subtle characteristics of tumors.

**Fig 1:**
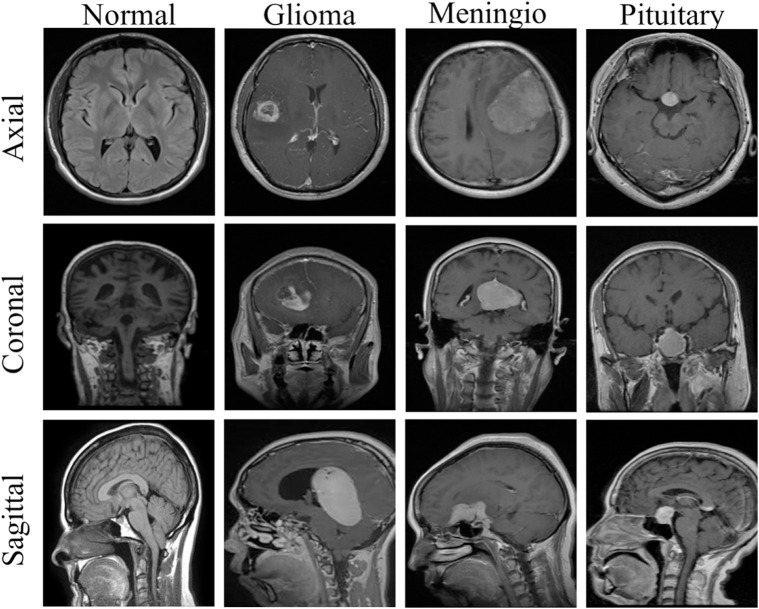
Different views and types of Brain MRI [8].

There have been many studies proposed to automate the tumor diagnosis using advanced machine learning and deep learning techniques [9–11]. However, these automated techniques face several challenges as well. First, tumors often have irregular shapes and a diverse composition [12], making it difficult to delineate clear boundaries and differentiate them from surrounding healthy brain tissue. As a result, there can be variability in interpreting tumors, causing inconsistencies in diagnoses and treatment plans. Second, imaging inaccuracies and discrepancies can occur due to various imaging device limitations. Finally, different views of MRI, including axial, coronal, and sagittal, introduce diversity and complexity in diagnosis as each provides distinct perspectives that are crucial for comprehensive brain tumor diagnosis [13]. The axial view provides horizontal cross-sections of the brain; however, its complexity in spatial resolution and the potential for overlapping structures can obscure tumor boundaries. The coronal view, which presents a front-to-back slice, poses challenges to in-depth perception and may require correlation with other views for a comprehensive assessment. The sagittal view, which provides a side profile, focuses on midline structures such as the corpus callosum and brainstem; however, interpreting these areas can be challenging due to their intricate anatomy and potential asymmetries. Fig 1 illustrates the different views of the brain MRI for four categories, such as normal, glioma, Meningioma, and Pituitary.

To address these challenges, we propose a potential solution, the View-specific Integrated Segmentation and Classification (VISION) model. This model combines a segmentation network with a classification header tailored for specific MRI views, utilizing specialized models to process different types of neuroimages. By doing so, we enhance diagnostic accuracy, improve consistency in tumor segmentation, reduce variability, and ensure uniform results. The proposed framework consists of three novel approaches. First, we employ view classification to accurately identify different MRI views. This ensures that the subsequent segmentation process can be optimized for specific orientations to address anatomical details, enabling precise localization and detection. Second, we have selected a pre-trained U-Net architecture as the backbone of our segmentation network and fine-tuned it using our dataset to effectively address data limitations for segmentation. Finally, we have integrated the segmentation model with a classification header to enhance its capabilities in detecting tumors and identifying their boundaries. Thus, we have proposed a novel brain tumor segmentation and detection framework by offering more accurate and specialized models for detecting and quantifying brain tumors compared to existing tumor detection methods. Therefore, the proposed framework provides decision support for caregivers by enhancing diagnostic speed and precision, highlighting potential tumor regions, and offering detailed tumor masks. We believe that our model will contribute to future advancements and further research in the field.

Therefore, the notable contributions of this research are outlined as follows:

- We propose and develop a view classification model that can automatically classify an input MRI into one of the three views, namely, axial, coronal, or sagittal, so that the corresponding VISION model can be used for tumor diagnosis. Thus, we address the complexity in brain tumor diagnosis caused by the diversity of MRI views.
- We systematically evaluate various pre-trained encoder backbones of U-Net to select the best encoder architecture for our U-Net segmentation model and fine-tune the selected encoder to enhance the accuracy and effectiveness of the segmentation model.
- We integrate a dedicated classification header with the fine-tuned U-Net segmentation model to detect the presence of a tumor and identify tumor boundaries, facilitating precise tumor diagnosis.
- We perform extensive experiments on benchmark datasets, showcasing improved segmentation and classification performance relative to current methods, thereby demonstrating the robustness and reliability of the proposed brain tumor diagnostic system.

The following sections of this paper are organized as follows: The Related works section provides a comprehensive review of related work, emphasizing key advancements and identifying existing gaps in brain tumor detection, segmentation, and MRI view classification. The Methodology section outlines the proposed methodology, detailing the theoretical background, system design, and computational models used. The Experimental setup section presents the experiments conducted to validate the proposed approach, including the experimental setup, parameters, and results, along with a detailed analysis. The Results and analysis section focuses on the ablation study, examining the effects of various modules within the proposed model. Finally, the Conclusion section concludes the paper by summarizing the main findings, highlighting the contributions, and suggesting directions for future research.

## Related works

The following section summarizes existing approaches for brain tumor detection and MRI segmentation. We explore related research in three main categories: **brain tumor detection, brain tumor segmentation, and MRI view classification**. Each category is discussed in detail in the subsequent subsections.

### Brain tumor detection

Several significant research contributions have been made on brain tumor detection and segmentation using deep learning models such as Convolutional Neural Networks (CNNs), Long Short-Term Memory networks (LSTMs), and Generative Adversarial Networks (GANs). The study [14] introduces a novel approach with a three-dimensional deep autoencoder network aimed at unsupervised anomaly detection in brain MRIs. This research focuses on training the model with normal brain MRIs rather than abnormal ones, utilizing a dataset of 578 normal T2-weighted MR volumes. The autoencoder-based model achieved at least a 7% improvement in accuracy compared to previous methods. However, the study did not consider the differential structure of the human brain. Incorporating advanced MRI segmentation techniques could strengthen the model’s performance in detecting abnormalities by providing more detailed insights.

Vankdothu et al. in the study [15] provide evidence on the challenges radiologists face, emphasizing the importance of tumor detection and advanced imaging techniques. Their model combines CNNs and LSTMs, achieving an accuracy of 92%. However, the study lacks sufficient evidence to support this claim, leading to ambiguity regarding the actual improvement. In addition, it does not include a detailed and efficient segmentation strategy. Incorporating MRI segmentation techniques could offer a more thorough solution, improving the model’s understanding of complex tumor structures. This study utilized a dataset of 3264 MRI slices of brain tumors, offering a decent number of cases for analysis. The CNN approach, which is discussed in the study [16], is utilized for the multistage classification of brain tumors, achieving notable accuracy. This model consists of three stages: differentiating between tumor-bearing and non-tumor MRI slices, categorizing tumor types, and determining tumor grades. The use of fast bounding box techniques for tumor segmentation is a key aspect of this approach. Similarly, another study [17] employs a multilevel classification strategy using CNNs, with both studies focusing on identifying and categorizing different levels of tumor severity. However, both studies show limitations in identifying tumor boundaries. The automated machine learning methods [18] for anomaly detection in medical imaging highlight significant benefits but also share the common limitation of focusing solely on detection without addressing segmentation.

The study [11] analyzes Convolutional Neural Networks (CNNs) and CNN-based deep learning models for the detection of brain tumors from MRI scans. Results show that EfficientNetB4 outperforms other considered models with an accuracy of 95%. The focus of the study was limited to classification alone, not ensuring the detection of the segmentation boundary. The convolutional auto-encoder network and 2D Convolutional Neural Network (CNN) were proposed in the article [19] for classifying brain tumors. Both of them attained a test accuracy of around 92%, which is quite a satisfactory result. Whereas, the study [20] evaluates the performance of three classification models and identifies that VGG16 and ResNet-50 secured the top two positions in terms of accuracy. Hence, we developed a hybrid model with VGG16 and ResNet-50, which improved the result even better. The performance of Convolution Neural Network (CNN) and self-defined Artificial Neural Network (ANN) is analyzed in the study [21] for the detection of brain tumors in MRI slices. Similarly, the article [22] emphasizes the effectiveness of a structured CNN in reliably detecting brain tumors from MRI scans, showing improved performance by optimizing parameters.

However, these works focus solely on the detection or classification of healthy or tumor MRI without determining the tumor boundaries. Hence, incorporating segmentation techniques could substantially boost the accuracy and performance of these models in medical imaging.

### Brain tumor segmentation

The DeepSeg framework, as outlined in [23], presents a generic decoupled architecture demonstrating effectiveness in tumor segmentation. It showcases a strong application of the UNet model. The study combined various deep learning models within a single system for feature extraction, but fell short in fully automating the system. The paper acknowledges that some existing methods are more efficient than the proposed model. However, it identifies several limitations, such as a concentrated focus on specific tumor boundaries, difficulties with heterogeneous brain structures, and a lack of discussion on computational efficiency. Addressing these issues by integrating efficient MRI segmentation algorithms could potentially enhance the accuracy and adaptability of the model. The interactive framework discussed in [24] is noted for its user-friendly tumor detection, but lacks clarity on the characteristics of the dataset. It used UNet and UNet++ for 2D and 3D segmentations, achieving dice scores between 94-97%. Considerations of potential biases and the need for validation in diverse clinical settings are essential. Improving this framework with advanced MRI segmentation techniques could boost accuracy and robustness in clinical applications. Therefore, precise segmentation will be crucial in advanced medical setups, such as telesurgeries, in the future.

The study by Sailunaz et al. [25] successfully utilized UNet for accurate segmentation without aggressive data augmentation, achieving good results when provided with tumor data. The study [26] utilized the U-Net model integrated with the FPN structure to capture multiscale contextual information. This was achieved by leveraging the multiscale features inherent in the U-Net architecture and the broad receptive field, advanced features provided by the FPN convolutional neural network, thereby enhancing the model’s flexibility in handling features of varying scales. The performance is good, with a satisfactory Dice rating and Jaccard index. However, the study [27] proposed Res-SegNet, Seg-UNet, and U-SegNet, which is a hybrid of the architecture, SegNet5, ResNet18, and U-Net. These proposed hybrid models were experimented on tumor data to achieve higher accuracy. Another hybrid approach was presented in the research [28] for brain tumor segmentation that integrates CNNs with manually extracted features. This method outperformed individual approaches that rely solely on handcrafted features or CNNs. However, despite its promising outcomes, the hybrid approach has certain drawbacks. For instance, combining hand-crafted features with CNNs can be complex and may require significant tuning for optimal performance. Furthermore, the method relies on extensive, labeled datasets for training, which can be difficult to access, especially in healthcare.

The article [29] provided an effective approach by exploring the Improved Residual Network (ResNet) for brain tumor segmentation. This method leverages a skip connection, where the initial input data is combined with the output of the convolution layers. This strategy helps mitigate the vanishing gradient problem by providing an additional path for the gradient to propagate through the network. Despite good results, the model did not consider segmentation as detection. The article [30] presented an upgraded version of the Mask RCNN model by integrating SENet’s channel attention and CANet’s spatial attention, which could help the model better recognize and extract informative features and, therefore, enable the model to capture and enhance tumor features in MRI images. However, the study [31] introduces a hybrid architecture, CiT-Net, utilizing the convolutional neural network parallel to vision transformers for medical image segmentation. Segmentation models worked for accurate segmentation over tumors of different shapes and sizes, but these models failed to give masks to the normal brain. Hence, segmentation cannot justify tumor detection.

### MRI view classification

Recent breakthroughs in deep learning and neural networks have shown encouraging outcomes in automating the classification process [32], reducing the reliance on manual expertise, and increasing the precision and performance of medical diagnostics. Human-driven classification of MRI views requires significant time. It is also susceptible to errors as a result of the subjective aspects of the task and the dependence on the expertise of radiologists. In response to these challenges, several studies were conducted to automate this process. A study [33] highlighted the effectiveness of a CNN-based approach in brain tumor classification, achieving a great result. This model classified images as tumor or normal before applying a hybrid segmentation technique to accurately delineate tumor regions.

The study [34] explored the application of Vision Transformers (ViTs) for MRI analysis, specifically in the context of Alzheimer’s disease (AD) diagnosis. ViTs, known for their capacity to capture long-range correlations in image data, were applied to classify MRI images into binary and multiclass categories. The proposed model outperformed traditional CNN-based methods, achieving an accuracy of over 99% in both binary and multiclass classification tasks. This study highlights the growing interest in leveraging advanced architectures like ViTs to enhance the precision and performance of MRI views and disease classification.

A comprehensive approach was presented in [35], where a deep learning model was developed to classify MRI images into 12 categories, encompassing different sequences (e.g., T1, T2, FLAIR) and views. The study utilized the MobileNet-v2 architecture, achieving a remarkable accuracy of 99.76% on unprocessed scans. The robustness of the model was further evidenced by its satisfactory performance on unseen data, with accuracy rates of 99.84% for online datasets and 86.49% for hospital data. This research underscores the importance of sequence and view classification as a foundational step in MRI analysis, facilitating the development of reliable computer-aided diagnosis (CAD) tools.

Table 1 summarizes the related works discussed in this paper. From the above literature review, we have identified the following research gaps in the existing works:

- Many studies concentrate solely on segmenting tumor images, which are trained only with tumor MRIs. This approach can lead to the misidentification of tumor masks in normal MRIs.
- Most approaches do not adequately consider the complexities introduced by diverse MRI views (axial, coronal, sagittal), overlooking the significance of anatomical spatial features for comprehensive and accurate tumor analysis.
- Many brain tumor detection and classification techniques do not include segmentation, making them unable to identify tumor boundaries.
- Finally, no study integrates and automates segmentation with classification for simultaneous tumor detection and accurate boundary localization.

**Table 1:** Summary of related works.

Study	Method Used	Key Findings and Limitations
Luo et al. (2023)	3D Deep Autoencoder	Achieved 7% improvement in accuracy but ignored brain structure differences.
Vankdothu et al. (2022)	CNN + LSTMs	Reached 92% accuracy, but lacks segmentation strategy and validation.
Aamir et al. (2022)	CNN Multi-stage Classification	Detects tumor presence, type, and severity but struggles with boundary identification.
Irmak et al. (2021)	CNN Multi-level Classification	Implements hierarchical classification but lacks effective tumor boundary detection.
Van et al. (2021)	Automated Machine Learning	Effective for classification but does not support segmentation.
Khaliki et al. (2024)	EfficientNetB4	Achieves 95% accuracy but is limited to classification.
Saeedi et al. (2023)	CNN + Autoencoder	Reaches 92% accuracy but does not focus on tumor boundary delineation.
Dhakshnamurthy et al. (2024)	VGG16 + ResNet-50 Hybrid	Improves classification accuracy but segmentation remains unaddressed.
Brindha et al. (2021)	CNN vs ANN	Compares CNN and ANN for tumor detection but lacks tumor boundary detection.
Martinez et al. (2024)	Optimized CNN	Optimizes CNN parameters for classification but does not provide segmentation insights.
Zeineldin et al. (2020)	DeepSeg (UNet)	Decoupled UNet, lack full automation and computational inefficiencies.
Sailunaz et al. (2023)	UNet, UNet++	Dice scores 94-97%, but dataset clarity is limited.
Walsh et al. (2022)	UNet without Augmentation	High segmentation accuracy but needs validation in diverse datasets.
Sun et al. (2023)	UNet + FPN	Multi-scale UNet with FPN for flexibility; lacks segmentation for normal brains.
Daimary et al. (2020)	Res-SegNet, Seg-UNet	Hybrid of SegNet5, ResNet18, U-Net; segmentation focus but lacks tumor detection.
Ullah et al. (2023)	CNN + Handcrafted Features	Combines CNN and handcrafted features; complex tuning required.
Aggarwal et al. (2023)	Improved ResNet	Skip connections mitigate vanishing gradients; no tumor segmentation.
Yuan et al. (2024)	Mask-RCNN + SENet + CANet	Improved feature recognition but lacks segmentation generalizability.
Lei et al. (2023)	CiT-Net (CNN + Transformers)	Hybrid CNN + Transformer but fails to provide masks for normal brains.
Valappil et al. (2021)	Deep Learning-based Classification	Reduces manual effort for classification but does not address view classification.
Singh et al. (2024)	CNN-based Classification	Achieves high classification accuracy but does not focus on segmentation.
Alp et al. (2024)	Vision Transformers (ViT)	Outperforms CNN in MRI classification but is computationally expensive.
Ali et al. (2024)	MobileNet-v2	Achieves 99.76% accuracy in 12 MRI view classifications; classification-only approach.

In this study, we address these gaps by proposing a view-specific model that integrates both segmentation and classification frameworks. This approach effectively utilizes MRI view classification, optimized encoder architectures, and integrated segmentation classification techniques to improve the accuracy of brain tumor diagnosis.

## Methodology

**Fig 2:**
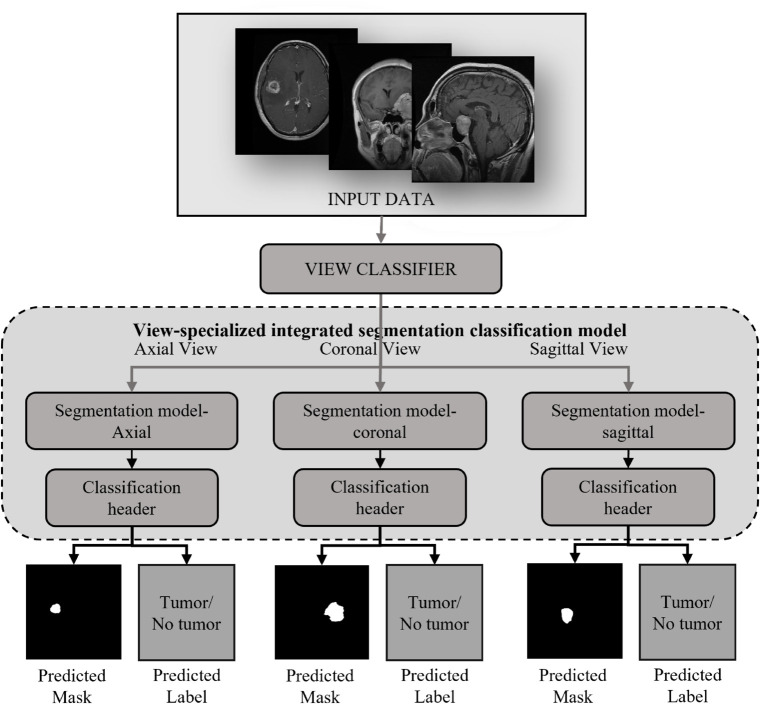
View-specific integrated segmentation and identification for optimizing NeuroDiagnosis.

**Fig 3:**
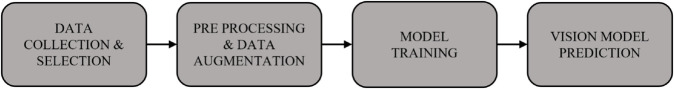
Main components of the proposed methodology.

In this study, we propose the View-specific Integrated Segmentation classification (VISION) model, a novel framework for tumor detection in Magnetic Resonance Images (MRI). The overall architecture of the VISION model is depicted in Fig 2. The framework employs a multi-view approach, where input MRI data is first classified into distinct views, followed by View-specific integrated segmentation-classification models to generate precise tumor masks and diagnostic labels for each orientation. The VISION framework is constructed through a systematic multi-stage pipeline, as illustrated in Fig 3. The pipeline comprises four critical stages: data collection and selection, pre-processing and data augmentation, model training, and the overall algorithm of the VISION model. Together, these stages optimize the precision and efficiency of tumor segmentation and identification. A detailed discussion of these stages is provided in the subsequent sections.

### Data collection and selection

We utilized openly available MRI datasets from kaggle.com to build three different datasets to make them suitable for all experiments. First custom dataset which is named **‘Encoder Fine-tuning Dataset’(EFD)**, comprising normal and tumor brain MRI with its corresponding masks, was constructed using the brain tumor segmentation dataset [36], which has 3064 tumor images and its masks and the normal Brain MRIs from the Brain tumor MRI dataset [37]. The dataset[37] consists of 8764 images of both tumor and normal brain MRIs, of which 3904 are tumor images and 3586 are normal brain MRIs. The images in this dataset lack masks to annotate the tumor, hence tumor MRI was not considered for any of our experiments. However, 3064 random images from the normal MRI were included in the EFD dataset to keep the dataset balanced. We provided a black image as the masks for normal MRIs, highlighting that normal MRIs will not have any tumors to be masked. The balanced EFD consists of 6128 images and 6128 masks, upon which the encoder was fine-tuned. The second dataset, **‘Main Working Dataset’(MWD)**, was formed by 4238 MRIs and their masks, which were taken directly from Kaggle [8]. The Main Working Dataset has four subsets: glioma (650 images), pituitary tumors (999 images), meningioma (994 images), and no tumor (1546 images), with all images resized to 512 pixels in both dimensions.

By manual classification performed by experts, the dataset was modified for further experiments by subdividing the MRIs based on their views, such as axial, coronal, and sagittal. For our analysis, 80% of the dataset (3,383 images) was used for training, while the remaining 20% (846 images) was reserved for validation and testing. In the previous section, Fig 1 will give an idea of brain tumor images from various categories. From Table 2, a detailed breakdown of the number of images in different views—axial, coronal, and sagittal—for each tumor type can be analyzed. The third dataset, which is named **‘View Classifier Dataset’(VCD)**, was derived from the second dataset, MWD, to train the view classifier. The dataset consists of three classes, each representing magnetic resonance images from three different views, without including masks in the dataset. The class breakdown is as follows: axial with 1010 images, coronal with 965 images, and finally, sagittal with 963 images. All coronal and sagittal images from the main working dataset are used without any alterations, but only 1010 images from 2301 axial images are included to prevent a significant class imbalance. Understanding the MRI view diversity remains relevant throughout our study as this plays a major role in it. An Axial view is a horizontal plane that separates the brain into upper (superior) and lower (inferior) parts like viewing the body from the feet upward or the head downward. The Coronal view divides the body into front (anterior) and back (posterior) sections, like viewing the body from the front or back. This plane runs parallel to the x-y plane and separates the body into front and back parts. Coronal resolution depends on slice thickness and matrix size along the anterior-posterior and superior-inferior axes. The Sagittal view is a vertical plane that splits the body into left and right halves, parallel to the y-z plane, offering a side view. The resolution in this plane is influenced by voxel size and the number of slices, which can vary depending on the area being imaged. Each of these plane views offers key insights into different anatomical structures, aiding in both diagnosis and treatment planning.

**Table 2:** MRI categories and views distribution.

Dataset	MRI Categories	Axial	Coronal	Sagittal
**MWD**	Normal	1465	38	93
	Glioma	205	250	194
	Meningioma	331	343	325
	Pituitary	309	334	351
	**Total**	**2301**	**965**	**963**
**VCD**	Normal	165	38	93
	Glioma	205	250	194
	Meningioma	331	343	325
	Pituitary	309	334	351
	**Total**	**1010**	**965**	**963**
**EFD**	Normal	3064		
	Tumor	3064		
	**Total**	**6128**		

**Fig 4:**
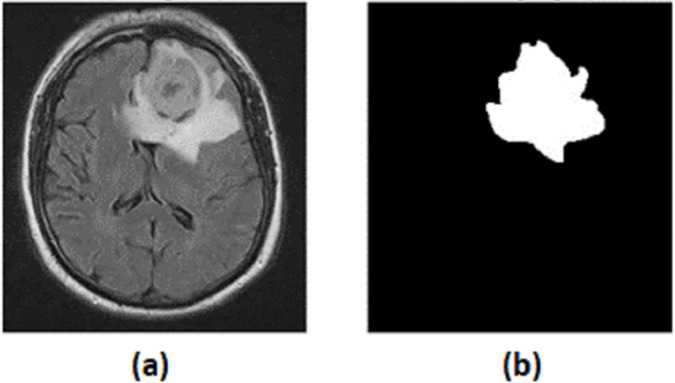
(a) Magnetic resonance image and (b) corresponding binary mask pair.

The dataset used for MRI segmentation includes MR images and their corresponding masks. Each data instance consists of a pair of MR images and its mask, as illustrated in Fig 4, where the MR image is the input data and its mask is the output or the segmentation. The incorporation of extensive labeled data (data available in open source platforms, which medical professionals curated) significantly escalates the predictive power of our model. A larger volume of training data contributes to building more robust models, and when data is limited, data augmentation methods can be employed to enhance the diversity of training samples, thereby improving the model’s generalization, which we will see in the next section.

### Data pre-processing and augmentation

Data pre-processing and transformation are fundamental phases in developing robust and accurate machine learning models, specifically in medical image analysis. Magnetic resonance images, especially those used to diagnose brain tumors, often come from diverse sources and vary significantly in terms of size, resolution, and quality. Without proper pre-processing, these inconsistencies can lead to challenges in model training, such as poor convergence, inaccurate predictions, and overfitting [38]. Therefore, standardizing the input data through pre-processing ensures that all images maintain consistent quality and resolution. This allows the neural network to process them effectively and focus on learning meaningful patterns rather than being distracted by irrelevant variations.

#### Resizing:

In our study, the pre-processing phase began by resizing all MRI images to a common size of 512x512 pixels. This phase was crucial to maintain consistency across the dataset, as the data images originally varied in dimensions, which could have led to difficulties in model training. By standardizing the image size, we ensured that the data fed into the model was uniform, enabling the model to concentrate on learning the essential features of brain tumors. Additionally, converting the images into a format compatible with the model’s requirements was an integral part of the preprocessing pipeline. This conversion ensured that the data was ready for efficient processing and analysis by the neural network.

#### Transformation:

Data augmentation enhances the generalizability and robustness of the model. In medical imaging, particularly with limited data samples, models are prone to overfitting, where they excel on training data but fail to adapt to unseen data. To reduce this risk, we applied several data transformation techniques to integrate variations in the images and mimic real-world scenarios. For view classification, the following transformations were applied during the training and validation phases.

#### Random rotation:

Random rotation introduces angular variations in images to improve robustness against different MRI orientations. An angle *θ* is randomly selected from a predefined range, typically $\left[-\theta_{\max},+\theta_{\max}\right]$. In our study, we set $\theta_{\max}=10^{\circ }$ and $\theta_{\min}=-10^{\circ }$. The image is rotated by this angle using the following transformation matrix:

$$\left(\begin{array}{c} x^{\prime }\\ y^{\prime } \end{array}\right)=\left(\begin{array}{cc} \cos(\theta ) & -\sin(\theta )\\ \sin(\theta ) & \cos(\theta ) \end{array}\right)\left(\begin{array}{c} x\\ y \end{array}\right).$$ (1)

Here, (*x*,*y*) are the image pixel coordinates, and $\left(x^{\prime },y^{\prime }\right)$ are the transformed coordinates. This ensures that the model becomes invariant to small angular deviations commonly encountered in real-world magnetic resonance imaging.

#### Random affine transformation:

The RandomAffine transformation applies a combination of rotation, translation, scaling, and shearing to the images. This helps the model to generalize better by simulating small positional and orientational variations in the MRI scans. The affine transformation can be mathematically described as:

$$\left(\begin{array}{c} x^{\prime }\\ y^{\prime } \end{array}\right)=A\cdot \left(\begin{array}{c} x\\ y \end{array}\right)+t.$$ (2)

Here, (*x*,*y*) are the image pixel coordinates, $\left(x^{\prime },y^{\prime }\right)$ are the transformed coordinates after the affine transformation, *A* is the 2×2 affine transformation matrix, and *t* is the translation vector that accounts for shifts in the *x* and *y* directions. Using RandomAffine transformations, the model learns to handle slight changes in the position and orientation of anatomical structures, improving its ability to classify views accurately.

### Model training

The study focuses on segmenting brain tumors from MRI images provided in .jpg format, using a two-stage approach that leverages the distinct characteristics of different anatomical views. In the Model training stage, separate models are trained for both view classification and segmentation, with an integrated segmentation classification model developed for each of the three primary views: axial, coronal, and sagittal. This view-specific approach for segmentation is based on the understanding that each view offers unique visual information and can yield more accurate and reliable segmentation results compared to a generalized model[39].

**Fig 5:**
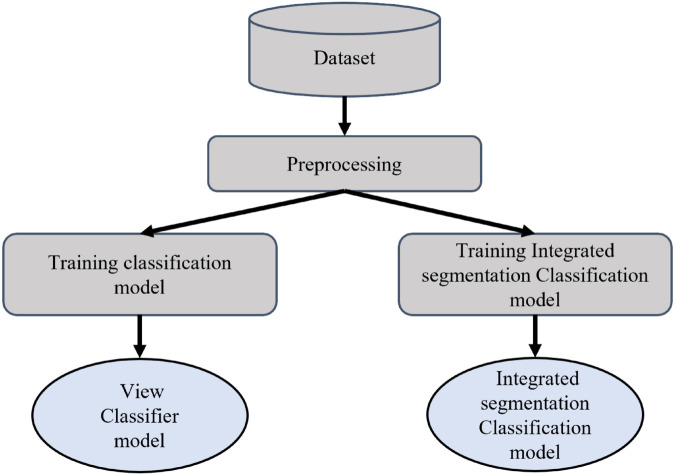
Model training flowchart.

Fig 5 gives a detailed idea of the entire model training process. The first part involves training a view classification model. This model categorizes the input MRI images into one of the three anatomical views: axial, coronal, or sagittal. Accurate classification is critical because it ensures that the appropriate view-specific segmentation model is selected in the subsequent step. The view classification model is designed to be lightweight and efficient, able to quickly determine the correct view, which is vital for the overall speed and performance of the system. Once the view classifier model is built, three View-specific integrated segmentation classification models are trained, each corresponding to one of the three views. These models are tailored to the specific features associated with each view. For instance, the axial view typically provides a cross-sectional slice of the brain, capturing a different aspect of the structure of the model tumor compared to the coronal or sagittal views. By training separate models for each view, the system can better capture relevant features and view-specific nuances, leading to more precise tumor segmentation. This approach is superior to using a generalized model, which may struggle to effectively segment tumors across all views due to the varying anatomical features and image characteristics present in each view.

#### Training view classifier model:

In terms of the details of the view classifier, we can say that it is a crucial component designed to categorize MRI images into three distinct anatomical views. This classification is essential to ensure that each MRI image is processed by the most appropriate segmentation model. The process of building the view classifier begins with data pre-processing, where MRI images undergo resizing, random rotation, random affine transformation, and normalization to prepare them for model training. The VGG16 model, which has been pre-trained, was selected for the development of the classifier due to its demonstrated efficacy in image classification tasks. The architecture of VGG16, comprising 16 layers, is particularly adept at extracting detailed features from magnetic resonance (MR) images [40].

By leveraging a pre-trained model, we reap the benefits from the extensive learning that VGG16 has undergone on large datasets, fine-tuned on our specific MRI dataset. The fine-tuning process involves adjusting the weights of the VGG16 model to optimize it for classifying MRI images into the correct view categories. This approach not only accelerates the training process but also improves the accuracy of the classifier, as it leverages the deep features learned by VGG16 during its initial training. The final output of this process is a robust view classifier capable of accurately categorizing MRI images into axial, coronal, or sagittal views. For the MRI view classifier, we use Weighted Cross-Entropy Loss (CE) during training to effectively manage multiclass classification, even with an imbalanced dataset distribution. The mathematical representation is given in Eq 3.

$$CE=-\sum_{i=1}^{C}w_{i}\cdot g_{i}\log(s_{i}),g_{i}\;\text{represents the ground truth.}$$ (3)

The **softmax** function is defined as:

$$s_{i}=\frac{\exp(o_{i})}{\sum_{j=1}^{C}\exp(o_{j})},\text{for each}i^{th}\text{output}\;o_{i}.$$ (4)

The weight is computed as:

$$w_{i}=\frac{Total\;number\;of\;samples}{Number\;of\;samples\;in\;class\;i},\;i\in \{1,2,\ldots ,C\}$$ (5)

#### Training integrated segmentation-classification model:

**Fig 6:**
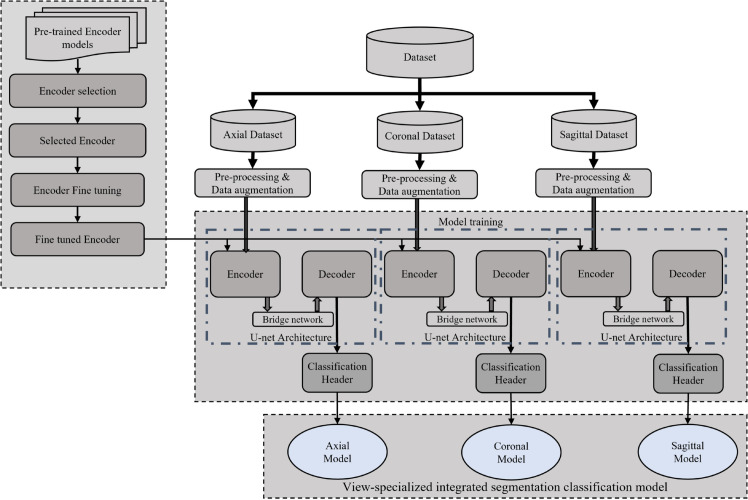
Training integrated segmentation-classification model.

**Fig 7:**
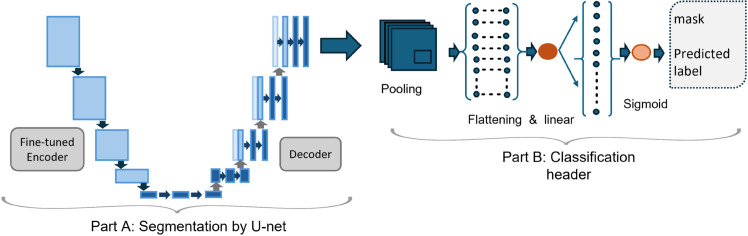
The proposed novel combination of tumor segmentation and classification model. It combines the fine-tuned U-net segmentation model with a classification header.

This stage is for training an integrated segmentation classification model for tumors from MRI images, which is shown in Fig 6. The proposed model training is performed through three sequential phases to systematically optimize feature extraction through encoder fine-tuning, building view-specialized segmentation models for distinct views to capture anatomical variances, and integration of segmentation and classification via a novel architecture. The process begins with the crucial task of encoder selection, which involves experimenting with various pre-trained models to identify the most suitable one for efficient feature extraction to avail precise brain tumor segmentation. Pre-trained models such as ResNet, EfficientNet, and InceptionNet are evaluated for their ability to extract meaningful features from the MRI images. The selected model is then fine-tuned as an encoder of the U-Net architecture as shown in Fig 7 Part A. U-net architecture consists of an encoder (to capture context) and a decoder (to enable precise localization), to perform the segmentation task. This fine-tuning process ensures that the encoder is optimized for brain tumor segmentation, enhancing its ability to identify and differentiate tumor regions from healthy tissue.

The View-Specific Segmentation Model Training phase is performed after the encoder fine-tuning. This step involves a specialized approach that leverages view-specific models tailored to the distinct anatomical perspectives of the axial, coronal, and sagittal views. Given the complexity of the MRI images and the unique visual characteristics presented by each view, a generalized model might struggle to handle the heterogeneous features of MRI. Hence, this approach addresses the limitations of generalized models, which are incompetent in capturing the intricate details required for precise segmentation in different views. Therefore, view-specific segmentation models are developed instead of relying on a single model, each fine-tuned to handle the specific challenges associated with its respective view. For each of the three views—axial, coronal, and sagittal—a dedicated U-Net model is trained. This specialized approach ensures highly reliable segmentation output, enabling precise differentiation between tumor tissue and healthy brain regions across the diverse and complex MRI datasets through view-specific processing guided by the view classifier.

The fine-tuned U-Net is extended with a classification header to predict tumor presence in the segmented tumor mask (Fig 7 Part B). The figure showcases our innovative architecture that enhances a fine-tuned U-Net segmentation model by integrating a specialized classification header. This addition facilitates critical diagnostic decision-making. This component analyzes segmented tumor regions to detect tumor presence in the MRI. The standard UNet focuses solely on pixel-wise segmentation, the enhanced UNet with a classifier header provides a global tumor presence prediction in addition to detailed segmentation, making it a more robust solution for medical diagnostics. The entire training is designed to optimize both the classification and segmentation processes.

To optimize the segmentation process, we implemented a custom loss function that incorporates Dice loss and Tversky loss to handle the intersection of prediction with ground truth and minimize the effect of imbalanced classes by introducing weighting parameters *α* and *β* to balance **false positives (FP)** and **false negatives (FN)** [41]. For imbalanced datasets, setting α>β gives more weight to FN, improving the segmentation of small objects.

$${ℒ}_{\text{Dice}}=1-\frac{2\sum (o\cdot g)+\epsilon }{\sum o+\sum g+\epsilon }$$ (6)

$${ℒ}_{\text{Tversky}}=1-\frac{\sum (o\cdot g)+\epsilon }{\sum (o\cdot g)+\alpha \sum ((1-o)\cdot g)+\beta \sum (o\cdot (1-g))+\epsilon }$$ (7)

where:

- *o* represents the predicted segmentation mask.
- *g* is the ground truth.
- ∑(o·g) is the intersection of the prediction and the target, which is true positive(TP).
- ∑((1−o)·g) is the false negative (FN).
- ∑(o·(1−g)) is the false positive (FP).

The final segmentation loss function is a **weighted sum of Dice and Tversky loss** 8:

$${ℒ}_{\text{seg}}=w{ℒ}_{\text{Dice}}+(1-w){ℒ}_{\text{Tversky}}$$ (8)

where *w* determines the priority of each loss.

To optimize the classification header, a suitable classification loss function is implemented, incorporating BCELogitloss with IoU loss to handle binary classification and to optimize spatial overlap between the predicted and ground truth regions as shown in Eq 11.

$${ℒ}_{\text{BCE}}=\frac{1}{N}\sum \left[\log(1+e^{-p})+o(1-t)\right]$$ (9)

To improve spatial accuracy, we integrate the **IoU loss**:

$${ℒ}_{\text{IoU}}=1-\frac{\sum (p\cdot t)+\epsilon }{\sum p+\sum t-\sum (p\cdot t)+\epsilon }$$ (10)

Where:

- *N* is the total number of pixels in each batch: N=batchsize×height×width
- *t* is the true label.
- *p* is the predicted label.

$${ℒ}_{\text{clas}}=\gamma {ℒ}_{\text{BCE}}+(1-\gamma ){ℒ}_{\text{IoU}}$$ (11)

where *γ* is a weighting parameter that balances classification and overlap losses.

Hence, both segmentation and classification processes play a vital role in the VISION model. The **total loss** for the model is a weighted combination of segmentation and classification losses, with respective weights as given in Eq 12.

$${ℒ}_{\text{total}}=(1-\lambda ){ℒ}_{\text{seg}}+\lambda {ℒ}_{\text{cls}}$$ (12)

where *λ* adjusts the influence of classification versus segmentation loss. This balanced optimization drives the model toward clinically actionable results such as precise tumor boundaries coupled with reliable tumor detection.

### VISION model overall algorithm

This phase acts as an automation of view classifier and tumor segmentation tasks. The model automation phase deploys the classification and segmentation models built in the model training phase to perform the tests. When an MRI image is provided, the view classification model first determines whether the image is axial, coronal, or sagittal, as shown in Eq 13.

$$v\leftarrow g(I_{p},M_{\text{clas}})$$ (13)

Where,

- *I*_*p*_ is the preprocessed image, representing the respective 2D slice.
- *v* is the class of images *I*_*p*_, indicating whether the MRI belongs to the axial, coronal, or sagittal.
- $M_{\text{clas}}$ is the weight learned during the training classification model.
- *g* is view classifier.

Based on this classification, the corresponding view-specific segmentation model is then selected to segment the tumor. For any view (axial, coronal, or sagittal), the goal is to predict the mask *S* to each pixel at (*i*.*j*) in the image *I*, with the segmentation model *f*, such that:

$$S[i][j]\leftarrow f(I_{p}[i][j],M_{\text{segclass\_v}})$$ (14)

$$p\leftarrow h(S,M_{\text{segclass\_v}})$$ (15)

where,

- *I*_*p*_ is the preprocessed image, representing the respective 2D slice.
- *S*[*i*][*j*] is the predicted mask for each pixel in (i,j) of image *I*_*p*_, indicating whether the MRI pixel belongs to healthy or tumor.
- $M_{\text{segclass\_v}}$ is the weight learned during the training of the segmentation model for each view, such that v∈{axial,coronal,sagittal} (Eq 13).
- $M_{\text{segclass\_v}}$ will be either $M_{\text{segclass\_axial}},M_{\text{segclass\_coronal}}\text{ or }M_{\text{segclass\_sagittal}}$.
- *f* is the segmentation for detection.
- *h* is the classification header function.
- p∈{0,1} representing healthy brain and tumor MRI.

The equation remains consistent across different views; however, the model’s design and training adapt to the specific orientation of the images. Additionally, the details of the model *f*, the receptive fields, and the management of spatial relationships depend on the image orientation. This automation ensures that the most appropriate model is assigned to each image, enhancing the accuracy of the segmentation process. This study also contributes to a more robust and reliable diagnostic tool, as each model is fine-tuned to address the specific challenges presented by its respective view. The study employs transfer learning techniques to mitigate the issue of limited data availability. The algorithm outlined in 1 for the proposed VISION model is designed to implement this approach by utilizing 2D MRI slices to identify their views for specific tumor detection. At the end of the process, a collection of segmented masks, M, is generated for detection.

## Experimental setup

This section will explain the experimental setup, results, and analysis.

**Algorithm 1** VISION: View-specific Integrated Segmentation classification model

**Require:** $D_{V}$: View classifier training data

1: *D*_*A*_: Axial view training data

2: *D*_*C*_: Coronal view training data

3: *D*_*S*_: Sagittal view training data

4: testD: Test data

**Ensure:** 𝒪: Tumor segmentation and detection output

5: **Preprocess**($D_{V}$, *D*_*A*_, *D*_*C*_, *D*_*S*_)   ⊳ Preprocess the input images

(Section Data pre-processing and augmentation)

6: $M_{\text{clas}}\leftarrow \text{trainViewClassifierModel}(D_{V})$   ⊳ Train view classifier

⊳ Train segmentation-classification models for each view

(Figure 7)

7: $M_{\text{segclass}[0]}\leftarrow \text{trainSegClassModel}(D_{A})$   ⊳ Axial view

8: $M_{\text{segclass}[1]}\leftarrow \text{trainSegClassModel}(D_{C})$   ⊳ Coronal view

9: $M_{\text{segclass}[2]}\leftarrow \text{trainSegClassModel}(D_{S})$   ⊳ Sagittal view

10: 𝒪←[]   ⊳ Initialize result list

11: **for all** image I∈testD **do**

12:   $I_{p}\leftarrow \text{Preprocess}(I)$

13:   $v\leftarrow g(I_{p},M_{\text{clas}})$   ⊳ Predicted view: 0 (axial), 1 (coronal),

2 (sagittal)

14:   $S\leftarrow f(I_{p},M_{\text{segclass}[v]})$   ⊳ Predicted segmentation mask (Eq. 14)

15:   $p\leftarrow h(S,M_{\text{segclass}[v]})$   ⊳ Predicted label (tumor/no tumor)

(Eq. 15)

16:   𝒪←𝒪∪{S,p}   ⊳ Store results

17: **end for**

**return** 𝒪

### System setup

The model was implemented using Python 3 within a Jupyter Notebook environment, leveraging PyTorch as the primary deep learning framework along with CUDA for GPU utilization. The training and evaluation processes were performed on a high performance computing system equipped with three NVIDIA A200 GPUs (12GB), which provided efficient parallel computation for deep learning tasks. The system was powered by an Intel Xeon Dual-Core Processor and 128GB RAM, ensuring smooth data loading, pre-processing, and large-scale MRI segmentation without memory bottlenecks. The VISION model occupies 3-4 GB of the GPU while the competing approaches utilize the remaining GPUs efficiently.

### Parameter setup

To enhance the efficacy of the training process, the AdamW optimizer [42] was utilized for both classification and segmentation tasks. This choice was made due to the optimizer’s adaptive learning rate and weight decay characteristics, which are advantageous in mitigating overfitting and promoting expedited convergence. The optimizer was assigned to minimize the BCEWithLogitsLoss for segmentation, to treat each pixel independently for pixel-level prediction, to examine the model’s precision region identification within an image. For the view classification model, crossEnropyLoss was minimized to handle multiclass classification.

The view classifier model was trained for 30 epochs, with a batch size of 16, a learning rate of 1e-5, and a weight decay of 0.05. The classifier’s effectiveness was critical for the overall system, as it ensured the correct segmentation model was applied based on the classified view. All experiments undergoing segmentation were conducted over 20 epochs with the same batch size of 3 and a learning rate of 0.001 to ensure fair comparison between competing approaches and to make it compatible with the machine.

The optimization parameters $w_{1},w_{2},\alpha ,\beta ,\gamma ,\text{ and }\lambda$ for training the VISION model are fine-tuned to obtain optimal values, which are given in Table 3. For the encoder selection process, the UNet model was trained to experiment with various pre-trained encoders to segment brain tumors in MRI images. The Encoder fine-tuning dataset, comprising 6,128 images, was preprocessed and utilized for experiment 2. The backbones or encoders involved in this experiment are ResNet, InceptionNet, and EfficientNetb0. The selected encoder is responsible for the further segmentation experiments, that is, experiments 3 and 4, which also followed a similar setup. The main working dataset, which contains 4238 images in 3 different anatomical views: axial, coronal, and sagittal, is used for the remaining segmentation experiments. In experiment 3, each view-based dataset comprises all tumor types in that specific view. Experiment 4 analyzes the threshold sensitivity as well as the influence of *λ* on the model.

**Table 3:** Performance metrics of different encoders.

Parameters	Value
*w*	0.4
*α*	0.7
*β*	0.3
*γ*	0.7
*λ*	0.2

### Dataset split

Three distinct datasets are used in this study, which are already discussed in Subsection Data collection and selection with a detailed description. **‘Encoder Fine-tuning Dataset’(EFD)**, **‘Main Working Dataset’(MWD)**, and **‘View Classifier Dataset’(VCD)** are used in experiments 1,2,3, and 4. The dataset was allocated in an 80-10-10 ratio for training, validation, and testing. The input channel configuration consisted of 3 channels, while the output channel was set to 1, with an input feature size of 512 x 512 pixels.

### Competing approaches

For this study we examined and compared four competing approaches for brain tumor segmentation, such as:

- Baseline U-Net [25]
- Optimized CNN [22]
- CiT-NeT [31]
- The VISION model (our proposed model)

Each of the approaches possesses a different complexity level, leaving room for assessing the impact of architectural modifications that influence segmentation performance. The baseline U-Net follows a traditional U-Net architecture with a symmetrical encoder-decoder arrangement. The encoder and decoder have several successive convolution layers. The Optimized CNN is a classification or detection model to classify tumors from normal brain using MRI slices. Based on the information given in the article [22], we were able to build the Optimized CNN. The third competing approach is CiT-NeT which use both CNN and transformers for segmentation. The code was partially available on GitHub. Utilizing the code available and other information from the article [31], we managed to conduct a comparison study with our model VISION. The VISION model implemented view-based classification prior to the segmentation task to ensure precise segmentation and handle data imbalance issues. The UNet segmentation part uses EfficientNetb0 as the backbone or encoder for the feature extraction process. These features were then given to the decoder for final segmentation. To ensure a fair comparison, all training and evaluation parameters are set to be the same. Even the data loader for training and testing for each competing model is the same.

## Results and analysis

### Evaluation matrices

Model evaluation involves calculating key metrics such as Intersection over Union (IoU), the Dice coefficient, F1 score, and accuracy. The F1 score provides a comprehensive view of the balance between precision, the model’s ability to accurately identify tumor regions without generating false positives, and recall, which measures how well it captures all true tumor regions without missing any. This score is essential for assessing segmentation quality, particularly in medical imaging, where minimizing both false positives and false negatives is crucial for an accurate diagnosis. In the context of segmentation, the IoU and the Dice coefficient are also significant. They are calculated on the basis of the concepts of union and intersection between the true mask and the predicted mask, using specific equations to measure the overlap between them. To convert the continuous value of the prediction, we used a threshold of 0.65, such that if *y*_pred_ or *p_i_* 16

$$p_{i}=\left\{ \begin{array}{cc} 1, & \text{if }\hat{y}\geq 0.65\\ 0, & \text{if }\hat{y}<0.65 \end{array}\right.\;\text{Hence, }p_{i},g_{i}\in \{0,1\}.$$ (16)

$$\text{IoU}=\frac{\text{Intersection}}{\text{Union}}=\frac{\sum_{i}p_{i}g_{i}}{\sum_{i}p_{i}+\sum_{i}g_{i}},$$ (17)

$$\text{Dice}=\frac{2\;\times \;\text{Intersection}}{\text{sum of prediction}+\text{sum of true}}=\frac{2\;\times \;\sum_{i}p_{i}g_{i}}{\sum_{i}p_{i}+\sum_{i}g_{i}}.$$ (18)

### View classifier and encoder selection

#### View classifier performance.

**Fig 8:**
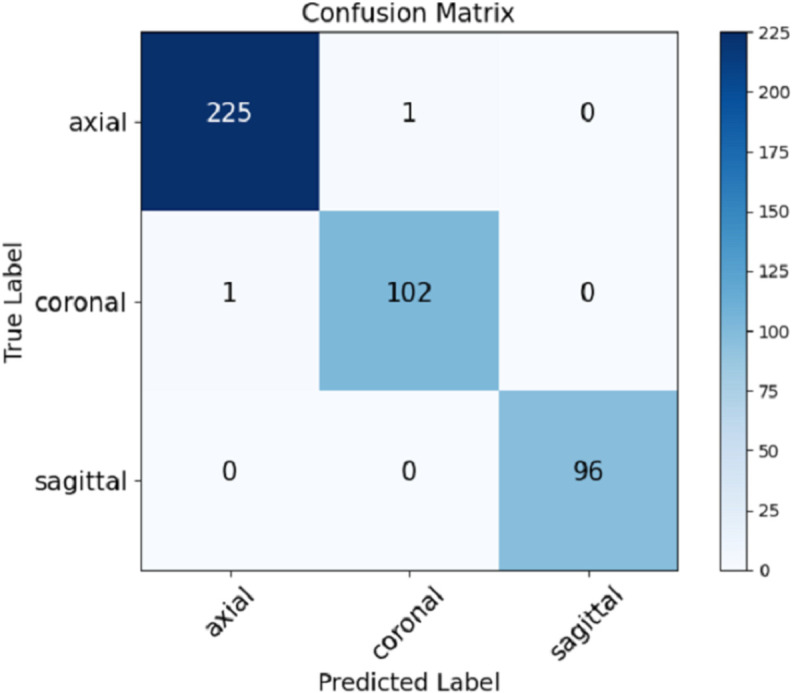
Confusion matrix for view classification.

The view classifier was trained to distinguish between axial, coronal, and sagittal views of MRI images. We tried two approaches for the view classifier, one with augmentation and the other without augmentation. Both approaches showcased excellent performance, but the view classifier trained with data augmentation achieved an overall accuracy of 0.99, while the one without augmentation scored 0.98. The view classifier with augmentation revealed that the classifier correctly identified nearly all images. The results we obtained assert that the model gives the best result, even if it has very few misclassifications. The precision, recall, and F1 scores for each view are relatively good, scoring approximately 99% throughout, which is well illustrated in the confusion matrix given in Fig 8. The high accuracy of the view classifier also ensures that the appropriate segmentation model can be applied based on the identified view.

#### Encoder selection.

In this experiment, various encoders were evaluated for their performance in segmenting the tumor in brain MRI images, which is listed in Table 4. **EFD** is used in this experiment, and the results were measured for the F1 score, the Dice score, and the Intersection over Union (IoU). The EfficientNetb0 encoder emerged as the best-performing model, achieving an F1 score of 0.7910, a Dice score of 0.8056, and an IoU of 0.8497. Other encoders, such as ResNet18 and ResNet34, also performed well, but EfficientNet outperformed them in all metrics. In contrast, InceptionNet showed significantly lower performance, with a Dice score of 0.5176. Therefore, the EfficientNetb0 encoder was used for experiment 3.

**Table 4:** Performance comparison of different encoders.

Encoder	F1 Score	Dice Score	IoU
ResNet18	0.724	0.748	0.826
ResNet34	0.726	0.753	0.831
ResNet50	0.714	0.750	0.831
ResNet101	0.648	0.699	0.731
ResNet152	0.728	0.713	0.770
InceptionNet	0.132	0.518	0.504
**EfficientNetB0**	**0.791**	**0.806**	**0.850**

### Comparison of competing approaches

This experiment focused on the performance of the segmentation model in different types of tumors (glioma, meningioma, pituitary) and views (axial, coronal, sagittal). The encoder selected from Experiment 2 was used in Experiment 3. The Table 5 illustrates a detailed comparison of the segmentation performances of the basic U-Net model, CiTNeT, Optimized CNN, and the VISION model in three different views: axial, coronal, and sagittal, along with all views combined with respect to evaluation metrics such as IoU, Dice score, F1 score, Precision, Recall, and Total Time (T). The values recorded are the average of results from five runs over random train-test-validation splits to ensure reliability of the results. Observing the performance of each model one by one, we can say that CitNet shows moderate IoU and Dice scores ranging from 0.4 to 0.65. Even took longer training times, such as for the largest dataset ’All view’, it took over 23,000 seconds, and for the smallest dataset, sagittal, it took over 5000 seconds. Test times are also relatively higher than those of other models. Interestingly, Recall often hits 1.000 in all cases, but precision varies widely (0.372–0.949). This suggests CitNet can capture all positives, but at times over-predicts, leading to lower precision in certain planes.

**Table 5:** Performance comparison among competing models.

Model	View	IoU	Dice	F1-score	Precision	Recall	T(s)
Vision	All view	**0.849** ± 0.028	**0.889** ± 0.025	0.970 ± 0.025	0.93 ± 0.03	0.96 ± 0.02	3284
	Axial	0.854 ± 0.026	0.889 ± 0.025	0.953 ± 0.024	0.93 ± 0.05	0.98 ± 0.03	1806
	Coronal	0.762 ± 0.023	0.833 ± 0.021	0.980 ± 0.005	1.00 ± 0.01	0.97 ± 0.02	705
	Sagittal	0.811 ± 0.039	0.873 ± 0.026	**0.984** ± 0.015	1.00 ± 0.01	0.97 ± 0.03	1090
CNN	All view	–	–	0.769 ± 0.009	0.83 ± 0.01	1.00 ± 0.00	1520
	Axial	–	–	0.545 ± 0.013	0.37 ± 0.01	1.00 ± 0.00	807
	Coronal	–	–	0.835 ± 0.005	0.95 ± 0.01	1.00 ± 0.00	297
	Sagittal	–	–	0.949 ± 0.010	0.90 ± 0.01	1.00 ± 0.00	327
CitNet	All view	0.630 ± 0.006	0.630 ± 0.003	0.769 ± 0.005	0.63 ± 0.00	1.00 ± 0.00	23597
	Axial	0.404 ± 0.005	0.486 ± 0.003	0.543 ± 0.006	0.37 ± 0.00	1.00 ± 0.00	12853
	Coronal	0.485 ± 0.003	0.524 ± 0.003	0.897 ± 0.005	0.95 ± 0.00	1.00 ± 0.00	5966
	Sagittal	0.444 ± 0.004	0.446 ± 0.002	0.876 ± 0.004	0.89 ± 0.01	1.00 ± 0.00	5417
Basic UNet	All view	0.645 ± 0.003	0.726 ± 0.002	0.726 ± 0.002	0.84 ± 0.00	0.84 ± 0.00	2885
	Axial	0.563 ± 0.003	0.696 ± 0.003	0.696 ± 0.003	0.80 ± 0.00	0.80 ± 0.00	1042
	Coronal	0.596 ± 0.003	0.713 ± 0.002	0.713 ± 0.002	0.82 ± 0.00	0.82 ± 0.00	494
	Sagittal	0.539 ± 0.003	0.625 ± 0.002	0.625 ± 0.002	0.77 ± 0.00	0.77 ± 0.00	831

An optimized CNN as a classifier could not produce a segmentation mask; as a result, the IoU and Dice scores were not recorded. However, performance metrics are reported such that F1 varies from ∼0.54 to ∼0.95, with higher F1 for Coronal and Sagittal than for Axial, and all views together show a moderate result. Precision follows the same trend, ranging between ∼0.37 and ∼0.94. Unfortunately, recall remains 1 over all planes. CNN is faster to train with ∼1,500 seconds, testing was also very quick, with fractions of a second to a few seconds. This indicates that the optimized CNN architecture is computationally efficient but exhibits inconsistent performance across different slice orientations. In contrast, the basic UNet achieves moderate performance for each of the MRI orientations and all views combined. The IoU and Dice score ranged between 0.5 and 0.75, providing balanced precision and recall, with a maximum of 0.84 and a minimum of about 0.76. This indicates more consistent detection without heavily favoring either sensitivity or specificity.

The maximum training time is reported for all views with ∼2,800 seconds, which is lower than CitNet but higher than the optimized CNN. It also sets a decent segmentation baseline that gives stable performance and moderate computational demands. The VISION model achieved the highest scores across most metrics. For all, the IoU and Dice score are reported as 0.84 and 0.88, and the F1 is around 0.96. The IoU for the views is recorded as 0.85 for axial, 0.86 for coronal, and 0.87 for sagittal. The Dice score also reported 0.88 for both axial and coronal and 0.89 for sagittal. F1 score is 0.93 for axial and ∼0.98 for coronal and sagittal. Precision and recall are also very high, indicating robust detection with minimal false positives or false negatives. The highest training time ∼3,176 seconds is higher than Basic U-Net but still much lower than CitNet, and the test time is moderate with ∼109 seconds. Hence, “Vision” performs best in terms of segmentation quality, although some models have higher computational costs.

**Fig 9:**
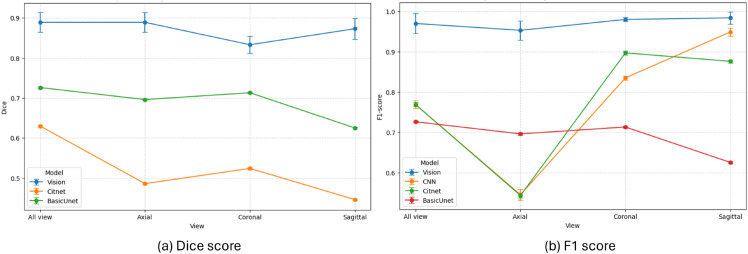
Summary plots for (a) Dice score and (b) F1 score.

We can conclude that the Vision model stands out in performance metrics. CNN is notably fast to train and test, Basic U-Net offers balanced performance, and CitNet shows high recall, but lengthy training times and poor segmentation results. These results provide a clear quantitative rationale for recommending Vision with maximum segmentation accuracy. With evidence of summary plot illustrated in Fig 9 we can observe the trend of Dice score (Fig 9(a)), and F1 score (Fig 9(b)) which indicates that the view-specific model strategy is better for brain tumor segmentation as well as classification, even with an imbalanced dataset.

To ensure the statistical significance of the observed performance differences, we also conducted paired t-tests between the Vision model and each baseline model. This analysis helps validate that the improvements are not due to random variations across multiple runs. All the entries considered for the t-test are listed out in Table 6. Table 7 presents the results of paired t-tests conducted to statistically compare the performance of the Vision model against three baseline models — CNN, Citnet, and Basic Unet — using the Dice and F1-score metrics. For all comparisons, the p-values are extremely small (all less than 0.00001), indicating that the differences in performance are statistically significant and unlikely to be due to random chance. Specifically, when comparing Vision to CNN and Citnet, the F1-score t-values are 6.2616 and 6.6872 respectively, while for Dice, they are 20.8784 and 20.8784. The comparison with Basic Unet yields even higher t-values: 25.3253 for F1-score and 15.4298 for Dice, highlighting a strong and consistent improvement. These results confirm that the Vision model significantly outperforms the baseline models in both segmentation (Dice) and classification (F1-score) accuracy.

**Table 6:** Dice and F1-score comparison across models over multiple runs.

View	Run	Vision		CNN	Citnet		BasicUnet	
		Dice	F1-score	F1-score	Dice	F1-score	Dice	F1-score
Axial	1	0.883	0.960	0.562	0.486	0.542	0.696	0.696
	2	0.885	0.926	0.550	0.489	0.550	0.693	0.692
	3	**0.930**	**0.989**	0.535	0.482	0.535	0.700	0.701
	4	0.862	0.953	0.548	0.488	0.548	0.695	0.695
	5	0.886	0.939	0.529	0.485	0.539	0.698	0.698
Coronal	1	**0.856**	0.973	0.836	0.524	0.899	0.713	0.713
	2	0.805	**0.984**	0.828	0.520	0.890	0.710	0.710
	3	0.820	0.978	0.841	0.528	0.902	0.716	0.716
	4	0.836	0.979	0.832	0.523	0.894	0.712	0.712
	5	0.850	**0.984**	0.839	0.526	0.898	0.714	0.714
Sagittal	1	0.867	0.989	0.947	0.446	0.876	0.625	0.625
	2	0.837	0.989	0.940	0.442	0.870	0.621	0.621
	3	0.865	0.959	0.962	0.448	0.880	0.627	0.627
	4	**0.899**	**1.000**	0.955	0.445	0.875	0.624	0.624
	5	0.898	0.983	0.939	0.447	0.878	0.626	0.626
All view	1	0.888	0.981	0.759	0.630	0.769	0.726	0.726
	2	0.892	0.986	0.762	0.645	0.762	0.723	0.723
	3	**0.928**	**0.992**	0.775	0.635	0.775	0.728	0.728
	4	0.862	0.933	0.781	0.631	0.771	0.725	0.725
	5	0.874	0.956	0.766	0.628	0.766	0.727	0.727
**Mean**		**0.871**	**0.972**	**0.774**	**0.522**	**0.771**	**0.690**	**0.690**
**± Std**		±0.032	±0.021	±0.151	±0.072	±0.144	±0.040	±0.040

**Table 7:** Paired t-test results comparing Vision with CNN, Citnet, and Basic Unet for F1 and Dice metrics.

Matrices	T test	Vision vs CNN	Vision vs Citnet	Vision vs Basic Unet
F1 score	t value	6.2616	6.6872	25.3253
F1 score	p value	0.000005	0.000002	0.000000
Dice	t value	–	20.8784	15.4298
Dice	p value	–	0.000000	0.000000

**Fig 10:**
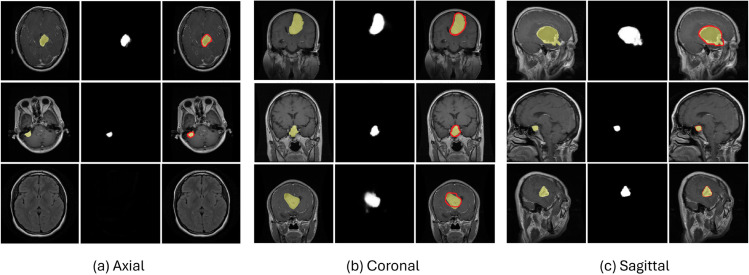
Segmentation results for (a) axial, (b) coronal, and (c) sagittal views.

The segmentation outcomes for the proposed view-specific model are pictured in Fig 10. In this figure, the tumor region is better envisioned by highlighting the true mask on the MRI (yellow), and the predicted tumor boundary (red) of the proposed model is given for better understanding and comparison. Fig 10a illustrates the segmentation of the axial view, which demonstrates notably high accuracy. The predicted tumor boundary is closely aligned with the ground tumor mask, demonstrating the model’s proficiency in accurately segmenting tumors. The true mask and the predicted mask show minimal discrepancy due to the model’s ability to analyze the anatomical features in the axial MRI slices. The segmentation of the coronal view shown in Fig 10b is less accurate than that of the axial view. There is a slight but noticeable divergence between the predicted boundaries and the true mask by either over-segmenting or under-segmenting the tumor in some regions. Similarly, the sagittal view is not as precise as the axial view in the segmentation process. The predicted boundaries generally follow the true tumor boundaries with a minor misalignment as in Fig 10c. The model struggles in these two views as they are vertical cross-sections displaying less tumor clarity and diverse anatomical information. As a result, the segmentation is less precise, resulting in the predicted mask and the ground truth aligning inconsistently, revealing gaps in between.

### Parameter sensitivity analysis

In this section, we will analyze the parameters *λ* and threshold to understand their effect on model training and model evaluation. The parameter *λ* plays a pivotal role in determining the optimization focus of the model. By increasing the value of *λ*, the total loss is primarily governed by the classification loss ${ℒ}_{\text{cls}}$. Whereas, keeping *λ* value intermediate, we can achieve a balanced approach, where both segmentation and classification losses contribute significantly to the total loss. And by lowering the *λ*, the total loss is predominantly influenced by the segmentation loss ${ℒ}_{\text{seg}}$.

**Fig 11:**
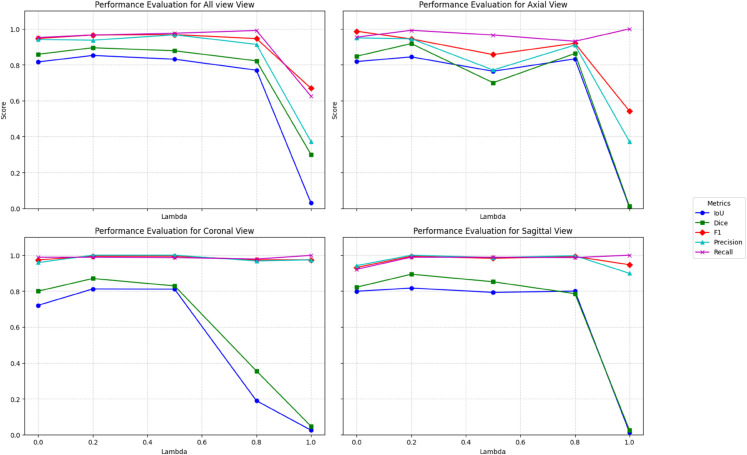
Effect of Lambda on model performance.

The graph displayed in Fig 11 likely reflects the impact of varying *λ* values (0.0 to 1.0) on the model’s performance metrics for various MRI views. The five key evaluation metrics (IoU, Dice, F1-score, precision, and recall) are plotted to assess segmentation accuracy and classification reliability. A notable observation is the sharp decline in performance at *λ* = 1.0, particularly in the IoU and Dice scores. This decline is most evident in the Coronal and Sagittal views, where the segmentation accuracy drops significantly. This suggests that when *λ* is set too high, the model struggles to balance segmentation and classification, leading to reduced spatial accuracy. At lower *λ* values (0.0 to 0.5), the model maintains consistently high performance across all views, with IoU and Dice coefficients remaining stable. The recall and precision scores remain close to 1.0 throughout all *λ* variations, indicating that the model is effective at identifying tumor regions, even when segmentation accuracy fluctuates. This suggests that while the segmentation quality varies with *λ*, the classifier’s ability to detect tumors remains relatively unaffected.

The Sagittal and Axial views demonstrate relatively stable performance across different *λ* values, indicating that the model adapts better to these orientations. However, the drop in IoU and Dice scores at higher *λ* values suggests that while model prioritizes classification over segmentation, the model may overemphasize classification confidence at the cost of fine-grained segmentation precision when the *λ* weighting is too high (e.g., λ≥0.8) which leads to significant performance degradation. The Recall and Precision metrics remain relatively unaffected, suggesting that tumor presence detection remains robust, even when segmentation quality declines. Based on these findings, the *λ* values should be carefully tuned, ideally within the range of **0.2 to 0.5**, to ensure a balance between segmentation accuracy and classification reliability. Also, the model can prioritize accurate segmentation, potentially at the expense of classification accuracy. We can also conclude that ***λ* = 0.2** allows the model to maintain high performance in both tasks, avoiding the pitfalls of overemphasizing either segmentation or classification at the expense of the other.

**Fig 12:**
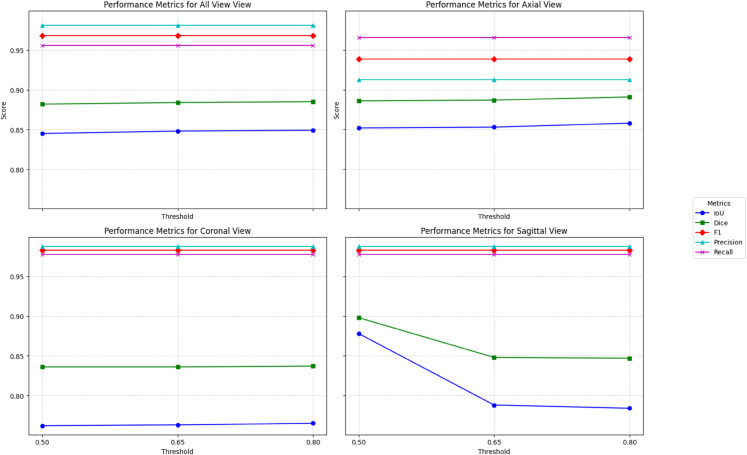
Effect of Threshold on model performance.

The segmentation model outputs continuous probability values between 0 and 1, which will not give a clear idea about the boundaries, as the intermediate probabilities (e.g., from 0.4 to 0.7) will not inherently indicate the classification decision. Hence, the threshold serves to convert this output to binary values 0 or 1 based on the specific cutoff. Selecting an appropriate ’threshold’ holds significant importance in the aspect of the segmentation task as it will determine the cutoff point for classifying pixel values belonging to the tumor region or not. By altering the threshold, we can evaluate its influence over the metrics, IoU, Dice score, F1 score, and accuracy, affecting the performance of segmentation by the VISION model. An apposite threshold will ensure a reliable and robust segmentation result, stabilizing the balance between false positives and false negatives. This will help in optimizing model performance by adjusting the decision boundaries for classifying pixels as tumor or non-tumor regions. For this experiment, we are considering three thresholds for evaluation, such as 0.5, 0.65, and 0.8, the results of which are illustrated in the graph shown in Fig 12. From the All View and Coronal View subplots, it is evident that performance remains relatively stable across different threshold values. IoU and Dice scores remain consistent, indicating that segmentation accuracy is minimally affected by threshold variations. Similarly, Precision, Recall, and F1-score maintain high values, suggesting that the model successfully detects tumors with minimal classification errors in these orientations. The stability across these views implies that the model is well-calibrated for detecting and segmenting tumors when considering multiple MRI planes.

The Axial View subplot exhibits a slight improvement in IoU and Dice scores as the threshold increases. This suggests that higher thresholds may slightly enhance segmentation accuracy in the Axial plane, allowing for better delineation of tumor regions by ignoring false positives. However, the Recall remains consistently high, reinforcing the model’s ability to correctly identify tumors even at lower thresholds. The stable classification performance alongside the slight segmentation improvement indicates that the Axial view benefits from a higher threshold without significant risk of misclassification. In contrast, the Sagittal View subplot reveals a notable decline in IoU and Dice scores as the threshold increases. This trend indicates that higher thresholds negatively impact segmentation accuracy in this view, leading to poorer tumor boundary delineation. Despite this, Precision, Recall, and F1-score remain stable, suggesting that while the model accurately identifies tumors, its ability to precisely segment them is compromised at higher thresholds in the Sagittal view. This sensitivity to threshold variation in the Sagittal plane suggests that an optimal threshold tuning strategy should be implemented to maintain segmentation quality. It is also evident that the varying threshold doesn’t influence the classification results.

### Ablation study

To evaluate the effectiveness of different components in our VISION model, we conducted an ablation study to systematically assess the contributions of the classification head, fine-tuning, and View-specific training strategies.

#### Study the effect of classification head and encoder fine-tuning:

We compared four variations of our model to understand the impact of the classification head and fine-tuning:

- **VISION**: Our full model with both segmentation and classification head.
- **VISION without classification head**: The model without the classifier head, focusing solely on segmentation.
- **VISION without fine-tuning**: The model trained without fine-tuning the encoder of the model.
- **VISION without fine-tuning & without classification head**: The model trained without fine-tuning the model.

Table 8 presents the quantitative results of our ablation study, comparing the Intersection over Union (IoU), Dice coefficient, and F1-score across different views. The results in the table indicate that the VISION model achieves the highest performance across all evaluation metrics. The IoU values for all views (0.845), axial (0.852), coronal (0.763), and sagittal (0.878) are the highest among all tested configurations. Additionally, the Dice coefficient and F1-score are consistently high, particularly in the sagittal view with Dice score 0.898 and F1-score of 0.983, and the coronal view, which reported 0.886 and 0.981 for Dice score and F1-score. This suggests that using segmentation enhanced with a classification head improves the model’s ability to generate precise tumor diagnoses.

**Table 8:** Ablation study results.

Method	View	IoU	Dice	F1-score
**VISION**	All view	0.845	0.888	0.968
	Axial	0.852	0.886	0.939
	Coronal	0.863	0.886	0.981
	Sagittal	0.878	0.898	0.983
**VISION w/o Classification Head**	All view	0.712	0.813	0.827
	Axial	0.895	0.897	0.830
	Coronal	0.778	0.844	0.885
	Sagittal	0.775	0.842	0.875
**VISION w/o Fine-Tuning**	All view	0.449	0.514	0.912
	Axial	0.399	0.433	0.802
	Coronal	0.486	0.584	0.942
	Sagittal	0.420	0.504	0.899
**VISION w/o Fine-Tuning & w/o Classification Head**	All view	0.580	0.657	0.664
	Axial	0.700	0.741	0.690
	Coronal	0.645	0.677	0.728
	Sagittal	0.634	0.688	0.692

When the classifier head is removed (VISION without classification head), performance drops noticeably, particularly in the IoU metric, dropping to 0.712 for all views. However, the axial view maintains a high IoU of 0.895, indicating that in some orientations, the classification head may not contribute as much. Despite this, the overall reduction in segmentation accuracy suggests that the classifier head aids in refining the final segmentation masks, leading to better agreement with ground truth annotations. The most significant drop in performance occurs when the model is trained without a classification head and fine-tuning. In this configuration, IoU for all views drops to 0.580, a 31% reduction compared to the VISION model. The coronal and sagittal views also show clear degradation in performance, emphasizing the importance of fine-tuning on medical imaging datasets. Similarly, the Dice coefficient and F1-score decrease across all views, highlighting that fine-tuning allows the model to adapt its pre-trained features to the specific domain, improving segmentation accuracy. The results in Table 8 demonstrate the impact of each model component:

- Removing the classification head leads to a significant drop in overall performance, with scoring IoU below 0.77, except for axial view with score 0.89. F1 score ranging between 0.82 and 0.89.
- Training without fine-tuning and classification head further degrades performance, with the IoU metric dropping to 0.58 for all views and the highest IoU reported 0.7, whereas the F1 score lies between 0.65 and 0.73.
- Without fine-tuning, performance remained moderate, with the Coronal view performing best in terms of classification but poor performance in segmentation, such that IoU=0.486 and F1=0.942), while the Axial view showed even weaker results (IoU 0.399, F1 0.802).
- The **VISION** model achieves the best performance across all evaluation metrics, demonstrating the benefit of both classifier integration and fine-tuning.

These findings demonstrate that both the classifier head and fine-tuning are critical for achieving optimal segmentation performance. The classifier head helps refine segmentation masks, leading to better precision, while fine-tuning ensures the model can effectively generalize to domain-specific features in medical images. The VISION model consistently outperforms the other variations, confirming that integrating both components leads to a precise diagnosis.

#### Study the effect of STSV and ATSV:

We further studied the effect of different View-specific training strategies on model performance based on single-type single-view (STSV) and all-type single-view (ATSV) approaches. In the Single Type Single View (STSV) approach, we consider each tumor type and each view independently. From Table 9 it’s evident that even though the approach gave significantly lower test results, especially giving poor IoU, dice score, and F1 score, which points out that the model is not suitable for brain tumor detection by the segmentation phase. Glioma coronal and glioma sagittal models gave scores in the range of 0.5 to 0.73, which is due to data inconsistency and insufficient data on glioma tumors. The second approach is All Type Single View (ATSV), where the model was trained on all tumor types but for a single view at a time. In Table 10, among the three single-view settings, the Sagittal view achieved the highest IoU (0.878), Dice (0.898), and F1-score (0.983), followed closely by the Coronal view, while the Axial view scored the lowest across all metrics. The results varied significantly across different views, with certain orientations showing improved stability and performance compared to STSV. This suggests that aggregating tumor types within a single view allows the model to learn more generalized features, partially mitigating the effects of data imbalance for individual tumor types. Overall, this ablation study highlights the importance of both architectural choices (such as the classification head and fine-tuning) and training strategies (favoring aggregated, multi-type datasets over isolated, single-type models) in achieving robust and precise tumor segmentation performance.

**Table 9:** Effect of Single Type Single View approach on VISION model.

Tumor Type	View	IoU	Dice	F1 Score
**Glioma**	Axial	0.922	0.936	0.750
	Coronal	0.524	0.638	0.591
	Sagittal	0.546	0.733	0.612
**Meningioma**	Axial	0.906	0.965	0.940
	Coronal	0.805	0.867	0.896
	Sagittal	0.827	0.864	0.943
**Pituitary**	Axial	**0.957**	**0.976**	0.929
	Coronal	0.834	0.906	0.905
	Sagittal	0.957	0.976	0.959

**Table 10:** Effect of All Type Single View approach on VISION model.

View	IoU	Dice	F1-score
Axial	0.852	0.886	0.939
Coronal	0.863	0.886	0.981
Sagittal	0.878	0.898	0.983

## Conclusion

This study aimed to address the significant challenges faced in the detection of brain tumors from MRI scans. These challenges include variability in tumor shapes, imaging disparities, radiological biases, and labor-intensive manual diagnosis, leading to inconsistencies in diagnosis and affecting patient outcomes. Hence, we propose system VISION, a View-specific Integrated Segmentation and Identification for Optimizing NeuroDiagnosis, to handle the variability of View-specific brain MRI by performing the view classification to employ the view-specific segmentation model with a classification head for tumor detection. We conducted several experiments to obtain the best results. We concluded that the view classifier achieves an average accuracy of ∼99% that leads to efficient utilization of MRI View-specific models. The EfficientNetb0 is the best-performing encoder among the other encoders tested, which is implemented in further experiments. The VISION model consistently delivered the best results throughout the experiments for each view in terms of both classification and segmentation. The results emphasize the prospects of our proposed model to substantially boost the reliability and efficiency of brain tumor detection through segmentation, showing promise for clinical use.
